# Somatic mutations in plasma cell-free DNA are diagnostic markers for esophageal squamous cell carcinoma recurrence

**DOI:** 10.18632/oncotarget.11409

**Published:** 2016-08-19

**Authors:** Masami Ueda, Tomohiro Iguchi, Takaaki Masuda, Yujiro Nakahara, Hidenari Hirata, Ryutaro Uchi, Atsushi Niida, Kota Momose, Shotaro Sakimura, Kenichi Chiba, Hidetoshi Eguchi, Shuhei Ito, Keishi Sugimachi, Makoto Yamasaki, Yutaka Suzuki, Satoru Miyano, Yuichiro Doki, Masaki Mori, Koshi Mimori

**Affiliations:** ^1^ Department of Surgery, Kyushu University Beppu Hospital, Beppu 874-0838, Japan; ^2^ Department of Gastroenterological Surgery, Graduate School of Medicine, Osaka University, Suita 565-0871, Japan; ^3^ Laboratory of DNA Information Analysis, Human Genome Center, Institute of Medical Science, University of Tokyo, Tokyo 108-8639, Japan; ^4^ Department of Surgery, Fukuoka City Hospital, Fukuoka 812-0046, Japan; ^5^ Laboratory of Functional Genomics, Department of Medical Genome Sciences, Graduate School of Frontier Sciences, University of Tokyo, Kashiwa 277-8562, Japan

**Keywords:** esophageal squamous cell carcinoma, cell-free DNA, next-generation sequencing, tumor recurrence, somatic mutation

## Abstract

**Objectives:**

Esophageal squamous cell carcinoma (ESCC) is one of the most aggressive malignancies owing to the high frequency of tumor recurrence. The identification of markers for early ESCC diagnosis and prediction of recurrence is expected to improve the long-term prognosis. Therefore, we searched for associations between tumor recurrence and cell-free DNA (cfDNA) mutations in blood plasma, which contains genetic markers for various cancer types.

**Experimental Design:**

Genomic DNA from tumors and cfDNA from plasma were obtained from 13 patients undergoing treatment for newly diagnosed ESCC. Next-generation sequencing of cfDNA in plasma was performed to identify mutations in 53 cancer-related genes, in which recurrent mutations were previously detected in ESCC. cfDNA mutational profiles were compared before and after tumor resection in four patients. Furthermore, somatic mutations in serial plasma samples were monitored after treatment in four patients.

**Results:**

We identified multiple concordant somatic mutations in cfDNA and primary tumor samples from 10 patients (83.3%) and in cfDNA and metastatic tumor samples from one patient (100%). Furthermore, the allele frequency of the concordant mutations in cfDNA changed concomitantly with tumor burden and increased approximately 6 months earlier than the detection of tumor recurrences by imaging tests in two patients. Conventional biomarkers, such as SCC and p53-Ab, did not reflect tumor recurrences.

**Conclusions:**

The present multigene panel, which enabled the diagnosis of tumor recurrence with greater accuracy than did using standard tumor markers or imaging methods, is expected to greatly facilitate standard, postoperative follow-up monitoring in ESCC.

## INTRODUCTION

Esophageal squamous cell carcinoma (ESCC), the most common subtype of esophageal cancer in East Asia, is one of the most aggressive malignancies owing to the high frequency of tumor recurrence [1, 2]. The identification of biomarkers for the accurate evaluation of tumor burden and early detection of tumor recurrence is crucial to ensure that effective therapy is administered in a timely manner [3].

Cell-free DNA (cfDNA) is present in the blood as small DNA fragments; these fragments can be released from dying tumor cells of not only primary tumors, but also metastatic tumors [4, 5]. Based on next-generation sequencing (NGS); droplet digital PCR; and beads, emulsion, amplification, and magnetics (BEAMing), cfDNA harbors genetic alterations associated with various malignancies [6–11]. Therefore, cfDNA, which may be obtained in a facile and non-invasive manner via “liquid biopsy,” is a potential source of diagnostic markers for the precise and early detection of ESCC.

In our previous study, we identified genes with recurrent mutations in Japanese ESCC patients by NGS [12]. Moreover, recent studies have provided important insights into the mutational landscape of ESCC and have identified recurrent mutations in driver genes [13–15]. The somatic mutations of such driver genes span large regions of the genome. Thus, it is difficult to examine mutations in these driver genes by PCR-based assays, and NGS analyses are more suitable for analyzing larger regions.

In this study, we constructed a panel of 53 previously identified driver genes in ESCC. We prospectively collected tumor and serial plasma samples and conducted NGS of the samples with the multigene panel. The aim of our study was to investigate the utility of NGS-based cfDNA analyses for the identification of clinically useful ESCC biomarkers.

## RESULTS

### Patient characteristics

**Figure 1:**
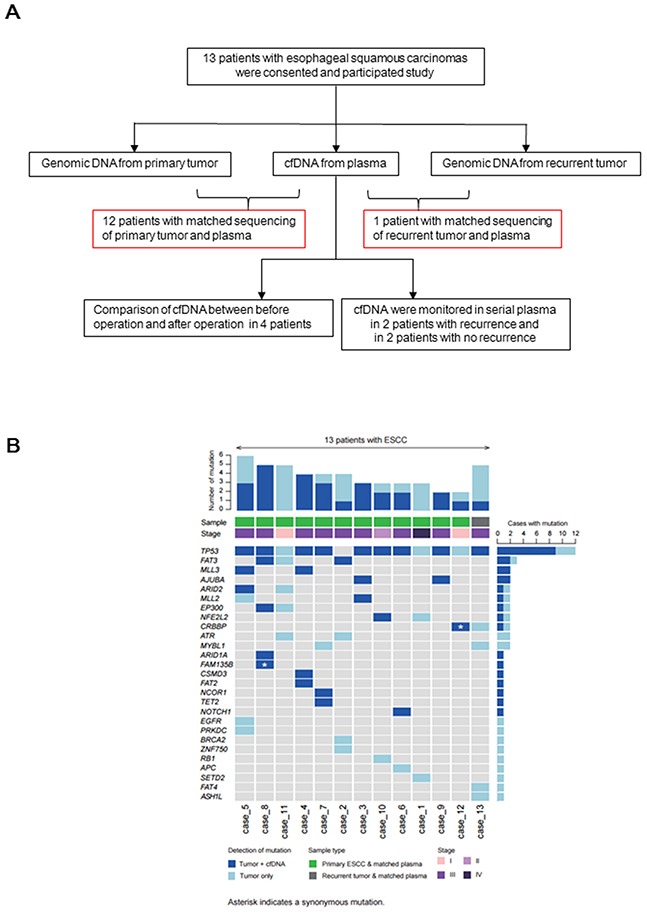
**A.** Study outline to assess the clinical utility of cfDNA in esophageal squamous cell carcinoma (ESCC); 13 patients participated in our study. Genomic DNA from primary tumors (12 patients) or a recurrent tumor (1 patient) and matched cfDNA samples were analyzed. Comparison of cfDNA before and after surgery in four patients; two patients with recurrence or no recurrence were monitored during follow-up. **B.** The number of somatic mutations in 53 genes in the tumor DNA and cfDNA samples from 13 patients (top), pathological (p)-stage and type of tumor sample (middle), and each mutated gene in the left column (bottom) are indicated.

The strategy adopted to assess the clinical utility of cfDNA in ESCC is shown in Figure 1A; 13 patients participated in our study. Genomic DNA from primary tumors (12 patients) or a recurrent tumor (one patient) and matched cfDNA samples were analyzed. cfDNAs from four patients (two with recurrence, two without) were obtained before and after surgery and monitored during follow-up. The clinical characteristics of 13 patients are shown in Table 1. We profiled 64 samples, including 12 primary tumor samples, 1 metastatic tumor sample, 38 plasma samples, and 13 matched normal tissue samples.

**Table 1:** Characteristics of 13 patients in our study

Patient number	Age	Sex	Location	p-Stage	T	N	M	Differentiation	ly	v	Residual tumor after surgery	NAC	TP53 status in primary tumor	Recurrence	Tumor source
1	60	Male	Lt	IV	3	1	1	Moderate	1	0	R0	DCF	Mutant	−	Primary
2	64	Male	Mt	III A	3	1	0	Poor	1	1	R0	DCF	Wild	−	Primary
3	79	Male	Lt	III A	3	1	0	Well	1	1	R0	DCF	Mutant	−	Primary
4	57	Male	Lt	III C	3	3	0	Moderate	1	1	R0	DCF	Mutant	+	Primary
5	66	Male	Lt	III B	3	2	0	Moderate	1	1	R0	DCF	Mutant	−	Primary
6	72	Male	Ut	III A	3	1	0	Moderate	1	0	R0	DCF	Mutant	+	Primary
7	77	Male	Mt	III B	3	2	0	Well	1	1	R0	DCF	Mutant	−	Primary
8	67	Male	Ut Mt	III A	3	1	0	Moderate	2	2	R0	−	Mutant	−	Primary
9	67	Male	Lt	III B	3	2	0	Moderate	1	1	R0	DCF	Mutant	−	Primary
10	48	Male	Mt	II B	2	1	0	Moderate	1	0	R0	DCF	Mutant	−	Primary
11	53	Female	Mt	I A	1	0	0	Well	1	1	R0	−	Mutant	−	Primary
12	64	Male	Mt Lt	I A	1	0	0	Well	0	0	R0	−	Mutant	−	Primary
13	66	Male	Mt	III C	3	3	0	Well	2	1	R0	DCF	—	+	Recurrent

NAC: Neoadjuvant chemotherapy

DCF: Docetaxel + Cisplatin + Fluorouracil

### Concordant somatic mutations between tumor samples and plasma samples

We examined the mutational profiles of 53 genes in primary and metastatic tumor samples from 13 patients (Figure 1B and Supplementary Table S1) and identified 56 tumor-specific somatic mutations (mean = 4.3 mutations per patient), including genes with recurrent somatic alterations, e.g., *TP53* (92.3%) and *FAT3* (23.0%). The *TP53*^H61L^ mutation was detected in only two patients. Next, we evaluated whether somatic mutations identified in tumor samples could be detected in the matched cfDNA samples. Twenty-nine somatic mutations detected in cfDNA samples were concordant with those in the primary or recurrent tumors (51.7%) (Figure 1B and Supplementary Table S1). We identified more than one concordant somatic mutation in cfDNA and primary tumor samples from 10 patients (83.3%) and in cfDNA and a recurrent tumor sample from one patient (100%). Among these, one concordant somatic mutation (*CREBBP*) was detected in a stage IA patient. Furthermore, to find more accurate biomarkers for identifying ESCC patients, we assessed the diagnostic utility of four genes (*TP53, FAT3, MLL3,* and *AJUBA*), mutations in which are the most recurrently detected. Mutations in these four genes were identified with 78.9% sensitivity, 100% specificity, and 92.3% accuracy in tumor and cfDNA samples from the patients (Table 2).

**Table 2:** Calculation of sensitivity, specificity, and diagnostic accuracy of cfDNA analysis for 4 genes

		Tumor		Sensitivity	Specificity	PPV	NPV	Accuracy
		Mutant	Wild	(%)	(%)	(%)	(%)	(%)
TP53	positive	9	0	75	100	100	25	76.9
cfDNA	negative	3	1					
FAT3	positive	2	0	66.6	100	100	90.9	92.3
cfDNA	negative	1	10					
MLL3	positive	2	0	100	100	100	100	100
cfDNA	negative	0	11					
AJUBA	positive	2	0	100	100	100	100	100
cfDNA	negative	0	11					
Total	positive	15	0	78.9	100	100	89.1	92.3
cfDNA	negative	4	33					

### Changes in the frequency of somatic mutations in cfDNA reflect changes in tumor burden

**Figure 2:**
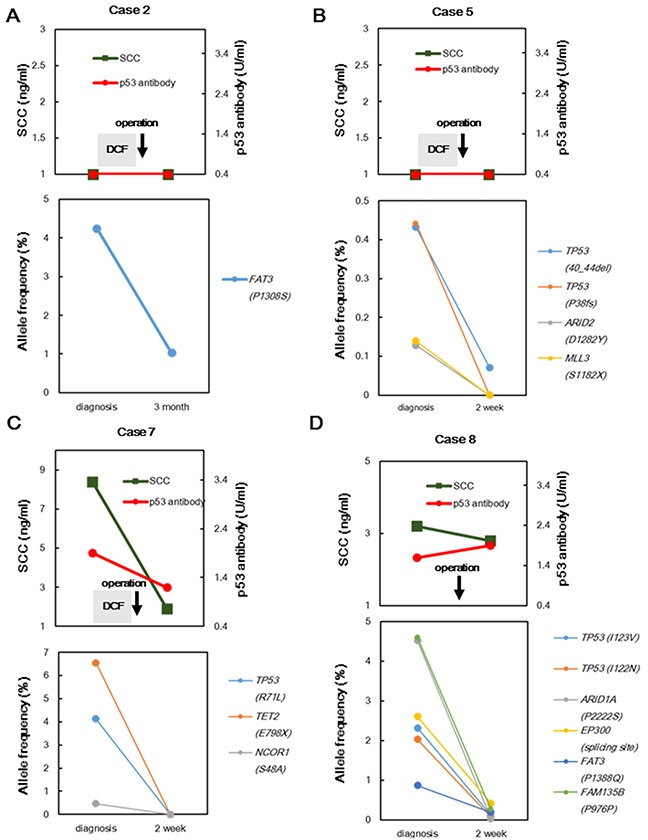
Comparison of AFs of concordant mutations before and after treatment by targeted sequencing of cfDNA from four patients; changes in conventional biomarkers and AFs of the concordant mutations after neoadjuvant chemotherapy and surgery in case 2 **A.** case 5 **B.** case 7 **C.** and case 8 **D.** SCC: squamous cell carcinoma-related antigen; DCF: docetaxel, cisplatin, and fluorouracil.

**Figure 3:**
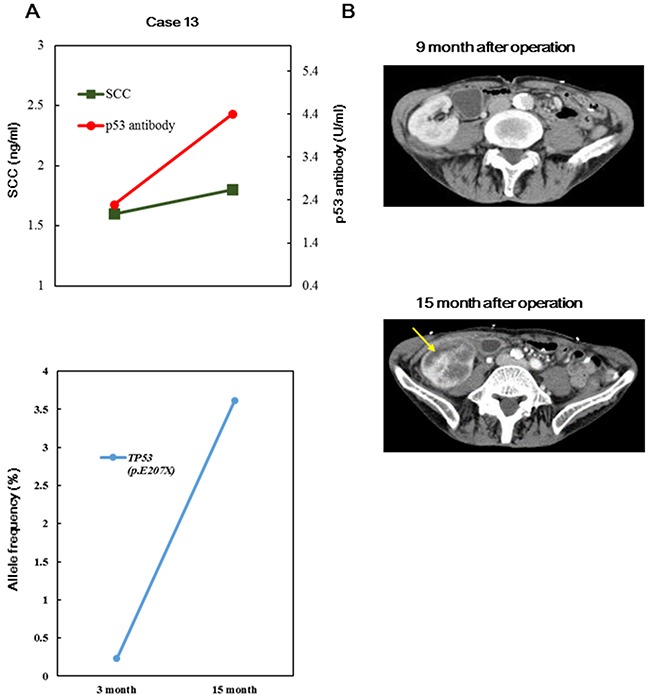
Comparison between before and after recurrence using targeted sequencing of cfDNA for case 13 **A, B.** Changes in conventional biomarkers, AFs of concordant mutations, and computed tomography (CT) findings before and after recurrence are indicated. Yellow allows indicate the recurrence of tumors in the kidney. SCC: squamous cell carcinoma-related antigen; DCF: docetaxel, cisplatin, and fluorouracil

To elucidate whether somatic mutations in cfDNA were quantitatively correlated with tumor burden, we monitored somatic mutations in serial plasma samples (Supplementary Table S2). First, we compared the allele frequencies (AFs) of concordant mutations identified in pretreatment cfDNA samples with those of mutations in post-treatment cfDNA in four cases. In four patients, the AFs of all concordant somatic mutations decreased remarkably (Figure 2). Next, we investigated the association between tumor progression and AFs in cfDNA. In case 13, the AF of the *TP53*^E207X^ mutation, which was concordant in recurrent tumor and matched cfDNA samples, remained extremely low until recurrence, at which point a 3.6% increase was observed (Figure 3). However, the concentration levels of cfDNA did not reflect either tumor reduction or tumor recurrence, which is consistent with previous findings [6]. These data suggested that concordant mutations in cfDNA change concomitantly with tumor burden.

### Monitoring somatic mutations in cfDNA for postoperative follow-up in ESCC

**Figure 4:**
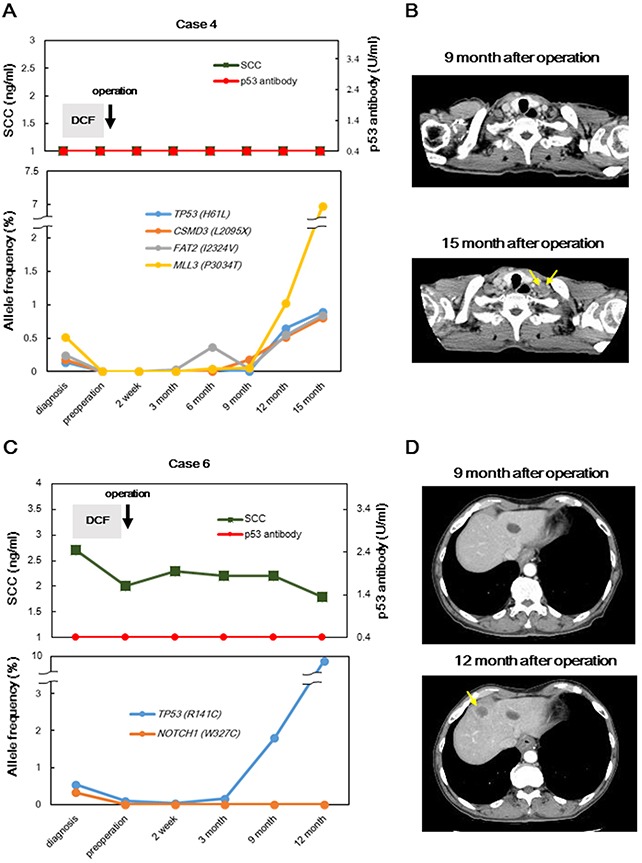
Disease monitoring in two patients with recurrences, from diagnosis to tumor recurrence, by targeted sequencing of cfDNA samples; changes in conventional biomarkers and AFs of concordant mutations and CT findings before and after recurrence in case 4 **A, B.** and case 6 **C, D.** are indicated. Yellow allows indicate recurrent tumors. AFs of non-concordant mutations were not detected in serial plasma samples in four patients. SCC: squamous cell carcinoma-related antigen; DCF: docetaxel, cisplatin, and fluorouracil.

**Figure 5:**
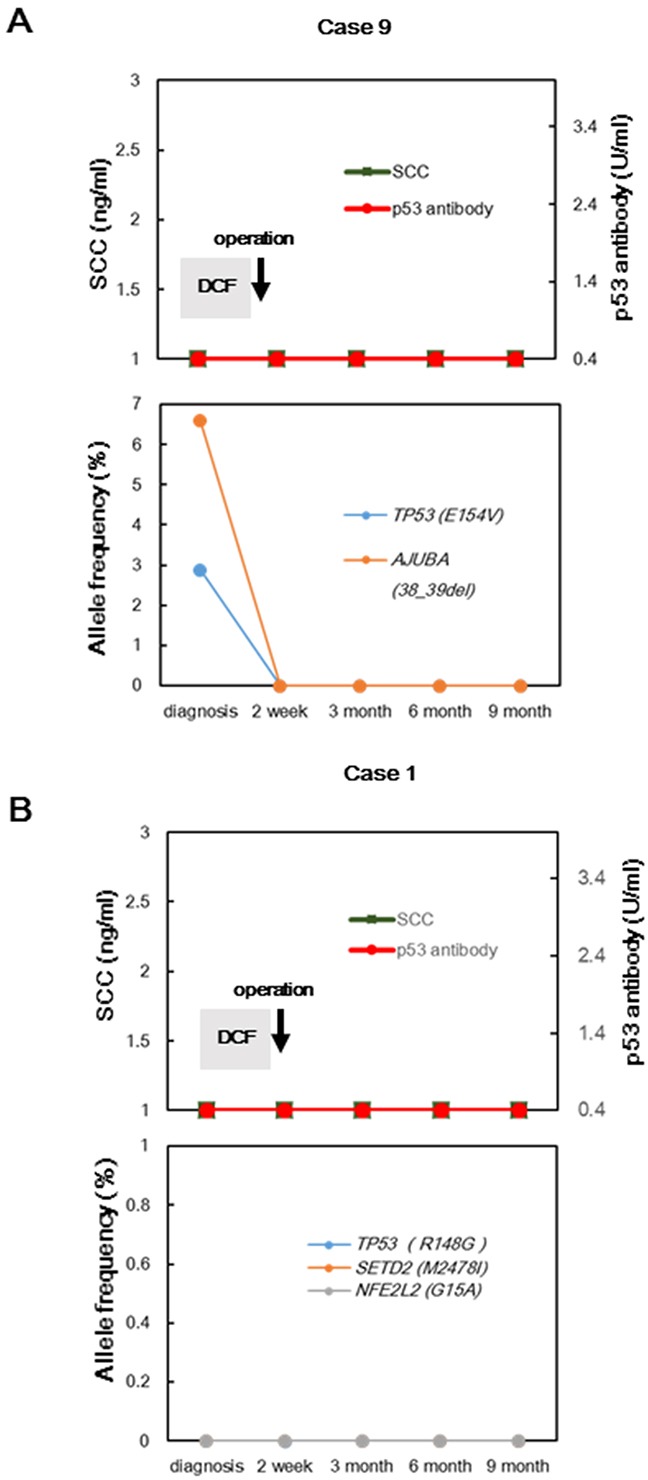
**A.** Disease monitoring in two patients with no recurrence during follow-up using targeted sequencing of cfDNA; changes in conventional biomarkers and AFs of concordant mutations for case 9; AFs of non-concordant mutations were not detected in serial plasma. **B.** Changes in conventional biomarkers and AFs of mutations detected only in primary tumor samples from case 1.

Next, we assessed whether cfDNA could provide clinically meaningful follow-up information for ESCC. We tracked serial samples after treatment in four patients. During the follow-up period, two patients (cases 4 and 6) had tumor recurrences, whereas two patients (cases 1 and 9) showed no recurrences. In case 4, SCC and p53-Ab were negative during the entire follow-up period and all concordant mutations were absent after treatment; however, the AFs of all concordant mutations increased 9 months before recurrence was detected by imaging tests (Figures 4A and 4B). Furthermore, in case 6, the AF of the *TP53*^R141C^ mutation decreased, and *NOTCH*^W327C^ was not detected after neoadjuvant chemotherapy and surgery (Figures 4C and 4D). Intriguingly, although the *NOTCH*^W327C^ mutation remained absent during follow-up, the AF of *TP53*^R141C^ gradually increased 6 months before hepatic metastasis was detected. SCC and p53-Ab levels remained within the normal range during the follow-up period. However, different results were observed in the two patients with no tumor recurrence. In one patient with non-recurrence (case 9), the concordant mutations did not increase in frequency during the follow-up period (Figure 5A). In the other patient with non-recurrence (case 1), somatic mutations derived from the primary tumor were also absent in serial plasma during the follow-up period (Figure 5B).

## DISCUSSION

In the current study, we applied NGS with a multigene panel to detect mutations in plasma samples in ESCC. Recent exome studies in ESCC have identified mutations in driver genes such as *TP53*, *FAT3*, *MLL3,* and *AJUBA*. Furthermore, plasma contains a very small quantity of cfDNA, which makes it difficult to detect mutant fractions emerging from malignant tissues. Therefore, we amplified cfDNA samples for NGS and focused on 53 genes; this approach enabled deeper sequencing coverage. In our cohort, 55 somatic mutations, but not *TP53*^H61L^, in tumor samples were detected in a single individual, indicating that NGS is adequate to examine mutational profiles in ESCC. This is consistent with the results of several recent studies using cfDNA [6, 10], suggesting that NGS with a gene panel can be used to effectively identify tumor-derived somatic mutations in various cancer types, using plasma samples.

In contrast to the study of other malignancies such as colorectal cancer, we found a clinical advantage in sequencing cfDNA from patients with ESCC to identify mutations in target genes. We previously observed diverse mutations in driver genes in colorectal cancer among patients with distinctive inter-tumor heterogeneity [16]. Therefore, it is difficult to establish a genetic panel for patients with colorectal cancer that is broadly informative. However, almost all patients with ESCC had mutations of one gene out of the top four genes (Table 2). We attained deep sequence coverage, which enabled us to accurately measure the tumor burden; therefore, using cfDNA is appropriate for ESCC cases.

We identified somatic mutations derived not only from primary tumors, but also from recurrent tumors, in cfDNA with high sensitivity and accuracy, suggesting that the genetic profiles of cfDNA can accurately reflect the status of tumors. Moreover, our analysis demonstrated that the AFs of concordant mutations in serial plasma samples are useful not only for evaluating the tumor status, but also for predicting tumor recurrence in ESCC. In particular, we demonstrated an increased frequency of concordant somatic mutations in cfDNA 6 months earlier than tumor recurrences were detected based on imaging tests in two patients. Similarly, Sausen et al. reported that mutations in cfDNA in pancreatic adenocarcinoma could be detected approximately 6 months before the detection of recurrences by imaging tests, which supported the utility of cfDNA analyses in ESCC in predicting tumor recurrences [11]. These data suggested that somatic mutations in cfDNA may reflect minor tumors that are not detectable by imaging tests and, accordingly, may provide a basis for the development of a diagnostic tool in ESCC. In this study, 1 concordant somatic mutation in *CREBBP* was detected in a stage IA patient. *CREBBP* mutations have been reported both in various solid cancers and in hematologic malignancies. In ESCC, *CREBBP* mutations and deletions of have been recurrently detected, and *CREBBP* acetyltransferase activities may be tumor suppressive [12–20]. *CREBBP* encodes a highly conserved and ubiquitously expressed nuclear phosphoprotein that belongs to the KAT3 family of histone/protein lysine acetyltransferases. *CREBBP*, together with the closely related protein *EP300*, also function as transcription factors involved in multiple signaling and developmental pathways by modifying lysine residues on both histone and non-histone nuclear proteins [21–24]. We propose that *CREBBP* might serve a fundamental function in ESCC tumorigenesis and that mutant *CREBBP* alleles might be frequently released into the bloodstream, as observed in the case of mutated *TP53*.

The AF of mutations in cfDNA was more strongly associated with tumor burden than conventional biomarkers of ESCC. Interestingly, in the two patients with tumor recurrence, the p53-Ab levels did not increase during recurrence, which was not consistent with the AF of the *TP53* mutation. Although p53-Ab is a circulating antibody against the mutated p53 protein in serum, its sensitivity is remarkably low according to a meta-analysis [25, 26]. In recent studies, it was demonstrated that ESCC is associated with various hotspot mutations in *TP53* [12–15]. In general, the p53-Ab is applied to treat tumors expressing mutated TP53 protein. The mutated *TP53* gene produces alternative epitopes in the variant TP53 proteins. In contrast, the limited epitopes recognized by the TP53-Ab cannot distinguish the variety of esophageal cancer cells with diverse TP53 gene mutations. These results suggest that the AFs of somatic mutations in cfDNA accurately reflect tumor status and may be superior to standard biomarkers currently used in ESCC.

Several mutations were detected in tumor samples, but not in cfDNA, and such mutations were absent in serial plasma samples from all patients. Furthermore, in case 6, although the *TP53*^R141C^ mutation gradually increased before tumor recurrence was detected, the *NOTCH*^W327C^ mutation remained absent during follow-up. We inferred that these results may reflect tumor heterogeneity. Heterogeneity exists within tumors and is an important factor underlying low therapeutic efficacy [27]. Tumors evolve by processes of branched evolution via the acquisition of genetic and epigenetic alterations, leading to the formation of various clones of cancer cells, including genetically distinct subclones, which contribute to tumor heterogeneity. Mutations detected in tumor samples, but not in cfDNA, may be derived from subclones of tumor samples and may rarely affect the formation of metastatic lesions. Thus, considering that tumor heterogeneity has an influence on tumor-derived mutations in cfDNA, it is important to investigate several driver genes, including *TP53*, related to ESCC in cfDNA. In addition, in case 1, we did not detect concordant somatic mutations despite the fact that the patient had stage IV ESCC. There is no definitive answer for this issue; however, we speculate that the outcome depends on the diversity of tumors in primary cases. According to the cancer-evolution model [16], greater diversity of mutations in primary tumors is observed in cancers in stage IV patients than in cancers from stage I–III patients. Therefore, the detectability of mutations in cfDNA derived from primary tumors might be reduced in patients with stage IV ESCC, compared to that in other patients. In addition, it is necessary to increase the accuracy of technologies used for cfDNA analysis.

In conclusion, although additional studies analyzing a larger number of samples are required, our findings suggest that NGS using a multigene panel is an effective method for detecting somatic mutations in plasma cfDNA. Our data support the use of cfDNA in clinical assessments of the tumor burden and suggest that cfDNA analysis may help predict tumor recurrence in ESCC.

## MATERIALS AND METHODS

### Ethics statement

Investigation has been conducted in accordance with the ethical standards and according to the Declaration of Helsinki and according to national and international guidelines and has been approved by the authors' institutional review board.

### Patient selection and sample collection

The study included 13 patients who were undergoing treatment for newly diagnosed ESCC between September 2013 and August 2015. The patients provided informed consent, and the study was approved by the Department of Gastroenterological Surgery (Osaka University) and the Department of Surgery (Kyushu University Beppu Hospital). All patients underwent tumor biopsy through an upper endoscopy. The diagnosis of primary ESCC was confirmed by histologic review. In 12 patients, tumor samples by biopsy were obtained from patients before they underwent neoadjuvant chemotherapy. For germline controls, adjacent esophageal normal tissue was obtained. In one patient (case 13), a biopsy sample was not collected because the primary tumor was too small. No patients received adjuvant chemotherapy following radical surgery. Plasma samples were routinely collected at the time of initial diagnosis, at the time of surgery in the operation room, at postoperative day 14, and at follow-up. p53 antibody (p53-Ab) and squamous cell carcinoma (SCC)-related antigen were measured simultaneously as conventional biomarkers [25, 28]. In three patients, tumor recurrences were identified by computed tomography tests. In 1 patient (case 13), the sample was collected from a recurrent tumor.

### Sample processing and DNA extraction

Plasma samples obtained from patients were collected in EDTA tubes. Plasma was centrifuged at 2500 × *g* for 10 min, added to microcentrifuge tubes, and further centrifuged at 16,000 × *g* for 10 min to remove debris. DNA was extracted from plasma with the QIAamp Circulating Nucleic Acid Kit (Qiagen, Hilden, Germany), per the manufacturer's protocol. DNA from tumor samples and the corresponding normal samples were extracted using the QIAamp DNA Mini Kit (Qiagen), following the manufacturer's protocol.

### Next-generation sequencing library construction

Indexed Illumina NGS libraries were prepared from plasma, tumor, and germline DNA. Plasma DNA was used for library construction without additional fragmentation. Tumor and germline genomic DNA were sheared before library construction with a Covaris S2 instrument (Woburn, MA, USA) to obtain 200-bp fragments. The NGS libraries of plasma DNA were constructed using the KAPA Hyper Prep Kit (Kapa Biosystems, Wilmington, MA, USA), following the manufacturer's protocols. A sequence library was prepared using a combination of the KAPA Hyper Prep Kit (Kapa Biosystems) and the SureSelect Target Enrichment System (Agilent Technologies, Santa Clara, CA, USA). End repair and A-tailing reactions were performed in 60-μL reaction volumes. The mixtures were incubated at 20°C for 30 minutes and 65°C for 30 minutes. Adapter ligation was performed using 110 μL and samples were incubated at 16°C for 16 hours using Agilent SureSelect Adapter. After post-ligation cleanup, the ligated fragments were amplified in 50 μL containing 2× KAPA HiFi HotStart ReadyMix and 10× KAPA Library Amplification Primer Mix. The following cycling protocol was used: 98°C for 45 s; 14–16 cycles (depending on the input DNA mass) of 98°C for 15 s, 65°C for 30 s, and 72°C for 30 s; and 1 cycle of 72°C for 5 min. Library purity, library concentration, and fragment length were determined using a 2100 Bioanalyzer (Agilent).

### Targeted massively parallel sequencing

Tumor, germline, and plasma DNA extracted from the samples of ESCC patients were captured using SureSelectXT Custom 1Kb-499kb, 16 (Agilent Technology), following the manufacturer's instructions. A panel of 53 genes was designed; recurrent mutations were previously identified in these genes, which are listed as potential driver genes in the Catalogue of Somatic Mutations in Cancer [12–15, 29] (Supplementary Table S3). The captured DNA was sequenced using the HiSeq2000 to generate paired-end (75–100 bp) reads for each sample. Targeted deep sequencing was performed for all samples using the multigene panel for a mean sequencing depth of 3810×.

### Mutation calling for genomic DNA from primary tumor and metastatic tumor samples

The sequence data for genomic DNA from tumor samples were processed using an in-house pipeline (http://genomon.hgc.jp/exome/). The sequencing reads were aligned to the NCBI Human Reference Genome Build 37 hg19 with BWA version 0.5.10 using default parameters (http://bio-bwa.sourceforge.net/). Mutation calling was conducted using the following parameters: (i) mapping quality score ≥ 25, (ii) base quality score ≥ 15, (iii) mismatched bases ≤ 5, (iv) both tumor and normal depths ≥ 100, (v) variant allele frequencies in tumor samples ≥ 0.05, (vi) variant allele frequencies in normal samples ≤ 0.05, and (vii) Fisher's exact test P-values < 0.05.

### Identification of mutations in plasma DNA

The sequence data for cfDNA from plasma samples were aligned to NCBI Human Reference Genome Build 37 hg19, following the same methods for genomic DNA obtained from tumors. Mutation calling was performed only at the positions with mutations detected in genomic DNA from tumor samples. The following parameters were used: (i) mapping quality score ≥ 25, (ii) base quality score ≥ 15, (iii) mismatched bases ≤ 5, (iv) cfDNA depth ≥ 100, (v) variant allele frequencies for cfDNA samples ≥ 0.0005, (vi) numbers of reads supporting mutation in cfDNA ≥ 2, and (vii) Fisher's exact test P-values < 0.05.

## SUPPLEMENTARY TABLES








