# Supplementary material for: Somatic mutations in plasma cell-free DNA are diagnostic markers for esophageal squamous cell carcinoma recurrence
Source: Oncotarget. 2016 Aug 19;7(38):62280–91. doi: 10.18632/oncotarget.11409 (PMC5308726; doi:10.18632/oncotarget.11409)
Supplement: Supplementary file 1 [file oncotarget-07-62280-s001.pdf]

Somatic mutations in plasma cell-free DNA are diagnostic markers for esophageal squamous cell carcinoma recurrence

SUPPLEMENTARY TABLES

Supplementary Table S1: Somatic mutations in tumor samples and matched cfDNA in plasma.

Supplementary File 1

Supplementary Table S2: cfDNA concentration and allele frequencies of concordant mutation in plasma and tumor markers in serum under surveillance in 13 ESCC patients.

Supplementary File 2

Supplementary Table S3: Comprehensive sequencing panel of 53 genes.

Supplementary File 3
